# Biophysical characterization of adeno-associated virus capsid through the viral transduction life cycle

**DOI:** 10.1186/s43141-023-00518-5

**Published:** 2023-05-17

**Authors:** Yanqiao Shawn Xiang, Gang Gary Hao

**Affiliations:** 1 Moderna Inc., 200 Technology Square, Cambridge, MA 02139 USA; 2 Weston Biomedical Reviews, 65 Autumn Road, Weston, MA 02493 USA

**Keywords:** AAV, Biophysics, Capsid, Endosome, Viral life cycle

## Abstract

Adeno-associated virus (AAV) vectors have emerged as the leading delivery platforms for gene therapy. Throughout the life cycle of the virions, the capsid vector carries out diverse functions, ranging from cell surface receptor engagement, cellular entry, endosomal escape, nuclear import to new particle packaging, and assembly. Each of these steps is mediated by exquisite structure features of the viral capsid and its interaction with viral genome, Rep proteins, and cellular organelle and apparatus. In this brief review, we provide an overview of results from over a decade of extensive biophysical studies of the capsid employing various techniques. The remaining unaddressed questions and perspective are also discussed. The detailed understanding of the structure and function interplay would provide insight to the strategy for improving the efficacy and safety of the viral vectors.

## Background

Adeno-associated viruses (AAV) are small (~ 26 nm) and nonenveloped viruses belonging to the family of Parvoviridae [1]. They have been broadly utilized in gene therapies for hereditary diseases such as neuromuscular, neurodegenerative, ocular, hemophilic, and lysosomal storage disorders [2–6]. Compared to other gene delivery platforms, such as lipid nanoparticle (LNP) and lentivirus, AAV vectors afford the advantage of high safety profile, lasting gene expression, broad tissue tropism, and capability of transduction of nondividing cells [7–10].

**Fig. 1:**
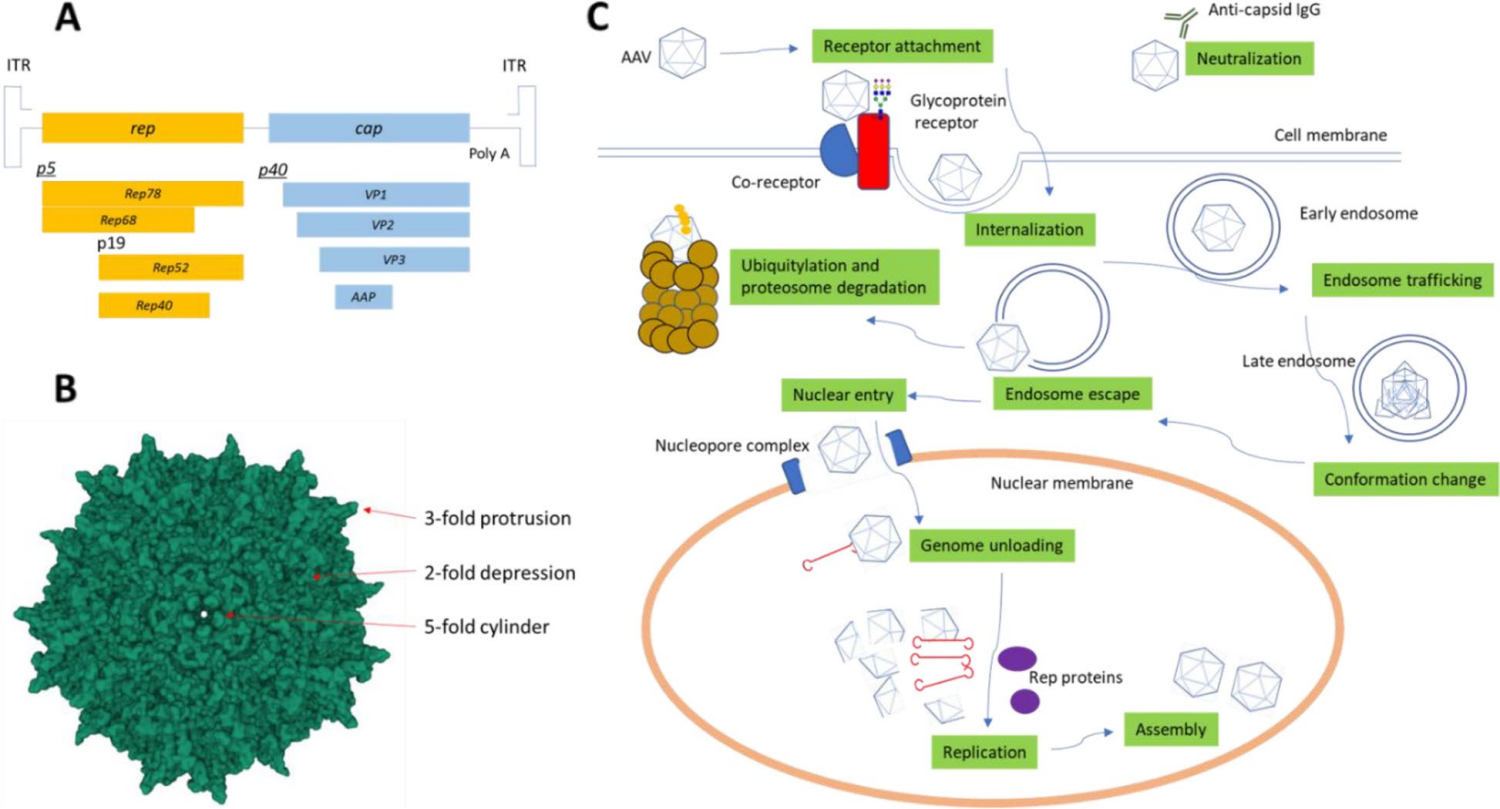
Schematic diagram for the transduction life cycle of AAV virions. **A** Genome structure of AAV. A single-strand DNA ~ 4.7 kb is flanked by two inverted terminal repeat (ITR). Multiple cap and rep genes are transcribed from two open reading frames through different promoters and alternative splicing. **B** Surface rendering of the crystal structure of AAV2. The capsid adopts an icosahedral conformation with key structural features including the fivefold channel, threefold protrusion, and the twofold depression. **C** Model of cellular entry and trafficking of AAV vectors. Following binding to a receptor/co-receptor complex, AAV enters target cell through endocytosis. Virions traverse through the trans-Golgi network including early and late endosomes. The conformation change in capsid exposes the phospholipase A2 (PLA2) domain to enable endosome escape and nuclear import via the nuclear pore complex (NPC). After nuclear import, intact capsids accumulate in the nucleolus followed by genome release in the nucleoplasm. The single-strand DNA is converted to double strand via the ITR and host-cell DNA polymerase. The rep and cap genes are then transcribed, and the new virion particles are assembled. The capsid can be also neutralized or eliminated by the antibody and ubiquitylation-proteasome system within the extracellular and cytosolic space, respectively

The genome structure and the transduction cycle of AAV are schematically depicted in Fig. 1. AAV carries a single-stranded DNA (ssDNA) genome of approximately 4.7 kilobases (kb) that encodes three open reading frames flanked by two inverted terminal repeat (ITR) [1]. The *cap* gene transcribes mRNA via the p40 promoter, which is further spiced into three viral proteins (VP1, 2, 3) [11]. VP1 spans the entire VP2 sequence in addition to a ~ 130-amino-acid N-terminal region, and the VP2 protein contains VP3 sequence in addition to a ~ 60-amino-acid N-terminal region. Sixty copies of proteins at ratio of approximately 1:1:10 for VP 1–3 assemble into the characteristic icosahedral capsid [11]. The assembly-activating protein (AAP) is also expressed from a frame shift of the cap gene [12]. The rep gene encodes four nonstructural rep proteins (Rep78, Rep68, Rep52, and Rep40) from the p5 and p19 promoter. The Rep proteins have endonuclease, DNA helicase, and ATPase activities that are essential for AAV DNA replication and packaging [13]. The inverted terminal repeat (ITR) adopts T-shaped motif, which functions as primer for double-stranded DNA synthesis inside the nucleus [14]. The cap and rep genes are replaced with the therapeutic gene of interest for AAV-based gene therapy [15].

The highly symmetric geometry of the viral capsids suggests a simple and static function of enclosing genome DNA. On the contrary, the capsid constitutes a dynamic entity with exquisite structural rearrangement and protein interaction to enable a range of functions throughout the viral life cycle. These include cell surface receptor recognition, endosomal trafficking and escape, nuclear entry, genome uncoating, viral replication and assembly, and immune neutralization and proteosome degradation [16, 17]. In this brief review, we summarize and discuss the current literature on the structural characterization of capsid during each phase. The structure–function understanding about capsid proteins will aid the development of AAV vectors with improved efficiency for gene therapy.

## Discussion

### Overall capsid structure

The capsid structures for multiple AAV serotypes have been solved by X-ray crystallography and cryogenic electron microscopy (cEM) [18–24]. The studies reveal highly conserved features across different serotypes, which includes a core comprised of eight-stranded antiparallel *β*-barrel and connecting loops. The 60 copies of the monomeric VPs assemble the icosahedral shell through two-, three-, and fivefold symmetric interactions. These interactions define the surface characteristics of the AAV, including depressions at the twofold (2F) axes, protrusions at the threefold (3F) axes, and cylindrical channels at the fivefold (5F) axes (Fig. 1B). The length of the connecting loops varies from a few to ~ 200 amino acids. Variable regions in the surface loops render distinct cellular tropism among different serotypes. Notably, only the conserved C-terminal VP residues are visible in the solved AAV structures, whereas the N-terminal sequences are not observed. The lack of defined N-terminal structure is likely due to the low copy numbers of VP1 and VP2 in the icosahedral assembly as well as the conformational flexibility. Interestingly, cEM studies of empty AAV2 capsids identified fuzzy density globules inside the capsid that was postulated as the N-terminal regions of VP1 and VP2 [25]. These globules were absent in mutant AAV2 structure where N-terminal parts of VP1 and VP2 were deleted [26]. In addition, a nucleotide binding pocket carrying an ordered nucleotide was also observed in the crystal structures of several serotypes, indicating the structural conservation of the genome anchoring region [20, 27].

### Capsid receptor complex structure

The transduction pathway of the AAV is initiated by the attachment to cell surface glyco-receptors. The primary receptors vary among AAV serotypes, including 2–3/2–6-N-linked sialic acid (SIA) (AAV1), heparan sulfate proteoglycan (HS) (AAV2, AAV3, and AAV6), 2–3 O-linked sialic acids (AAV4), 2–3 N-linked sialic acids (AAV5 and AAV6), and galactose (AAV9) [28]. The structures of AAV in complex with glycan receptors have been solved by crystallography and cEM [29–31]. The studies reveal that the 3F protrusion is the primary sites for glycan binding across the serotypes. AAV2 HS binding site is located to basic residues at the 3F protrusions. Additional conformation changes were observed at the 2F and 5F axes, suggesting capsid rearrangement occurring during the cellular entry. The SIA binding site for AAV1 was also located at the base of 3F protrusion. For AAV5, two SIA molecules interact with the depression of the 3F axis and the surface loop close to the 5F axis. AAV-DJ binds to heparinoid pentasaccharide at the right side of the 3F spike.

Importantly, recent studies identified a universal AAV coreceptor (AAVR) which is also required for AAV cellular entry in addition to the primary glycan receptors [32]. The AAVR receptor is a transmembrane protein comprised of five tandem polycystic kidney disease-like (PKD) domains (PKD1-PKD5). The structures of AAV complexed with AAVR have been solved by cEM for multiple serotypes [33–35]. AAV2, AAV1, and AAV9 bind to the PKD2 domain of AAVR, whereas AAV5 binds to the PKD1 domain. PKD2 resides on the plateau of the AAV2 capsid and interacts with the inner facet of 3F spike. In contrast, PKD1 contacts the outer rim of the 3F spike on the AAV5 capsid. The high-resolution structure reveals no long-range conformational change in AAVR upon AAV binding. Cryogenic electron tomography (cET) was applied to probe the conformation of the non-AAV engaging domains of AAVR [36]. For AAV5, the nonbinding PKD2 domain displays three configurations extending away from the virus. The PKD1 domain of AAV2 adopts four configurations of PKD1, all different from AAV5 interacting PKD1 conformation. Unlike other serotypes, AAV4 is unable to interact with AAVR, and its cellular co-receptor remains elusive. A comparison of AAV4 structure with AAV5, AAV2, and AAV1 in complex with AAVR explains the lack of affinity for the AAV4 clade [37].

### Capsid antibody complex structure

The clinical application of AAV as a gene therapy vector has been hindered by neutralizing antibody (Ab) induced from the humoral immune response, which often limit the clinical administration of AAV therapeutic to single dosage [8, 38]. cEM followed by 3-D image reconstruction have been used to define the epitopes for neutralizing antibodies against AAV1, AAV2, AAV5, and AAV8 [39–42]. The studies reveal significant conserved epitope footprint on capsid surface across the serotypes despite variations in the amino acid sequences. Ab A20 binds to AAV2 on the plateau between the 2F and 5F axes and the floor surrounding the 5F channel. Ab C37-B binds to 3F protrusions on AAV2, which overlaps with HS receptor binding site. Ab 4E4 and 5H7 bind to 3F protrusions of AAV1. Ab ADK8 targeting AAV8 also binds to the 3F protrusion. The structure of AAV5 in complex with four Abs has been reported, including two neutralizing Abs ADK5b and H2476 and two non-neutralizing Abs, ADK5a and 3C5. Ab ADK5a and 3C5s epitope partially overlap that of PKD1 at the floor of the 2F depression. Ab H2476’s footprint locates in the vicinity to the binding site of SIA near the 3-F protrusion, whereas the epitope for ADK5b resides near the 5F channel. These data provide structural basis for the neutralization effect including steric interference with receptor binding as well as post-cell attachment and pre-nuclear entry.

### Endosome trafficking and escape

Once the AAV enters the host cells via receptor-mediated endocytosis, it traverses through the endosomal system before breaking into the cytosolic milieu. The capsid undergoes conformational shift at acidic pH conditions within the late endosome, which exposes the N-terminal domain of the VP1. The phospholipase A2 (PLA2) activity of the N-terminal regions disrupts the lipid membrane leading to endosome escape of the viral particle [43, 44]. The capsid structures at different pH conditions throughout the endosomal system have been investigated by a variety of biophysical methods. The structure of AAV2 was determined by cEM and 3D image reconstructions at different pH conditions experienced in the extracellular space, early endosome, late endosome, and lysosome [45]. The study reveals conformation rearrangements of the variable loops accompanying the drop in pH, whereas the core structure remains constant. Differential scanning calorimetry (DSC) and small-angle scattering (SAS) analysis also indicate mild structural changes in response to pH shift. Small-angle neutron scattering (SANS) analysis confirmed a genomic rearrangement event that accompanied the capsid structural changes. Circular dichroism (CD) demonstrates that the VP1u is structurally ordered in solution as predominantly *α*-helical, whereas a gradual loss of secondary structure was seen with increasing temperature and/or decreasing pH [46]. When the pH was restored to 7.5, the secondary structure was restored to the original state. The crystal structures of green fluorescent protein (GFP) AAV8 have been determined at pH of 6.0, 5.5, 4.0, and 7.5 after incubation at pH 4.0, following the events during endosomal trafficking [47]. While the overall capsid topologies remain similar, significant amino acid side chain l motion was observed on the interior surface of the capsid near the ordered nucleic acid density. AAV9 capsid structure was determined across pH 7.4, 6.0, 5.5, and 4.0 in free as well as galactose-bound form at pH 7.4 and 5.5 using cEM and three-dimensional image reconstruction [48]. The study observed capsid conformational changes at the 5F channel during the externalization of the VP2/VP3 N termini. In addition, it shows that AAV9-glycan receptor complex remains intact at the late endosome pH 5.5. Taken together, these studies confirm the N-terminal externalization of VP2/VPs occurring at acidic pH and the conformation change at the 5F channels as a possible prelude to genome uncoating.

### Ubiquitylation and proteosome degradation

Within the cytosol, the capsid is subjected to ubiquitylation followed by proteasomal degradation [49]. The resulting peptides are presented by major histocompatibility complex (MHC) class 1 molecules to CD8 + T cells. The CD8 + T cell can exert destructive cytotoxic effects to eliminate rAAV-transduced cells, leading to the loss of transgene expression. Western blot of immunoprecipitated AAV2 and 5 capsid proteins from infected HeLa cell lysates revealed the presence of ubiquitin conjugation [48]. Mutation of the surface tyrosine, serine, threonine, or lysine residues significantly reduced ubiquitylation and enhanced AAV2 transduction efficiency [49–51].

### Nucleus entry

The AAVs enter the nucleus through the nuclear pore complex, which is believed to be mediated by the nuclear location signal in VP N-terminal region. The BC2 and BC3 domains shared by the N-terminus of VP1 and 2 are separated by a 23-amino-acid linker, which classifies them as a nonclassical nuclear localization signal, which confer nuclear localization to a heterologous fusion protein [52]. A high-speed super-resolution single-point edge-excitation sub-diffraction (SPEED) microscopy study revealed that AAV2 particles are imported through nuclear pore complexes (NPCs) rather than nuclear membrane budding into the nucleus [53]. Moreover, approximately 17% of the rAAV2 molecules starting from the cytoplasm successfully transverse the NPCs to reach the nucleoplasm, revealing that the NPCs act as a strict selective step for AAV delivery.

### Genome uncoating

The viral particle releases its genome content in the nucleolus. The physical process of genome ejection has been studied in vitro under thermally stressed condition [54] for AAV1, AAV2, AAV5, and AAV8 using differential scanning fluorimetry (DSF), differential scanning calorimetry (DSC), and EM showed that capsid melting temperatures differed by more than 20 °C between the least and most stable serotypes. The ultrastructural differences in genome release from AAV containing single-stranded DNA (ssDNA), or self-complementary DNA (scDNA), were compared by atomic force and electron microscopy [55]. The scAAV vectors required significantly higher thermal threshold than ssDNA. Genome release was also monitored by a fluorometric method, which demonstrated that acidic pH and high osmotic pressure inhibit genome release and an inverse correlation between the genome size and the uncoating temperature. In another study [56], the stability of AAV8 and AAV9 was profiled by atomic force microscopy (AFM) and physical modeling. The study indicates that genome release can proceed via two alternative pathways: intact capsid ejects linear ssDNA or ruptured capsid ejecting entangled ssDNA. To date, the exact cellular condition triggering AAV genome release remains elusive. It was postulated that the disassembly of AAV2 capsids is induced by the structural reorganization of the nucleolus in a cell cycle-dependent manner [57]. Interestingly, AAV2 virions are completely and rapidly dissociated following incubation with a liver nuclear extract [58]. More systematic investigation of the physiological conditions encountered by AAV in the nucleolus is required for the complete understanding of the genome uncoating process.

### Replication and assembly

Following the genome uncoating, the life cycle of the virus enters the reproductive stage including replication and assembly. While the therapeutic AAV construct is devoid of the self-replication and assembly functionality, understanding about such process is important for improving the production yield in manufacturing process.

Sucrose density sedimentation experiments showed that VPs oligomerize in the cytoplasm (trimers or pentamers), but do not form capsids [59]. In the nucleus, the vast majority of VPs is assembled into capsids with sedimentation coefficient between 60 and 110S. Mutational studies showed that the VP3 sequence is sufficient for the assembly of AAV capsids by knocking out the start codons of VP1 and VP2 [60]. The composition of several AAV serotypes was investigated by high-resolution native mass spectrometry [61]. The data reveal that the capsids assembly is a stochastic process, forming a heterogeneous population of capsids with variable VP stoichiometry. Specific residues important for the formation of the AAV capsid have been identified via mutagenesis of residues distributed over the entire VP1 sequence followed by characterization of their ability to abolish capsid assembly [62]. Residues located at the 2F and 5F interfaces of the VP and close to the 5F channel play important roles in capsid assembly. In addition to VP3, the viral cofactor assembly-activating protein (AAP) is also essential for capsid assembly [63]. AAP was reported to bind to the VP multimers across the 2F axis to facilitate capsid assembly. The role of AAP was evaluated in 12 naturally occurring AAVs [64]. The results demonstrate that AAP enhances capsid protein stability and interactions. Moreover, the study showed that the dependence on AAP can be partly overcome by strengthening interactions between VP monomers within the capsid assembly.

The genome packaging process occurs in the nucleus and relies on the presence and interaction of the assembled empty capsids, replicated AAV genome, and the Rep proteins. DNA encapsulation is directed by protein–protein interactions between empty capsids and complex of Rep78/68 with the virus genome. During genome replication, Rep78/68 remains covalently attached to the nascent ssDNA and docks on the 5F axis of the assembled capsid [65]. The Rep52/40 proteins are responsible for transferring the AAV genome DNA into empty particles through the 5F channel. The role of various capsid residues in genome packaging was investigated by site-directed mutagenesis [66]. Mutational residues around the 5F channel abolished Rep-capsid interaction and genome packaging. Mutation of a residue near the 3F axis also prevents packaging. This residue is however not exposed on the capsid surface but affects the stability of the capsid, suggesting that capsid stability is required to compensate for the increase in pressure during packaging. Conformational changes in AAV1 induced by genome packaging were studied by cEM that reveals conformational changes upon packaging of the genome [67]. The rearrangements occur at the inner capsid surface and lead to constrictions of the 5F channel.

## Conclusion

Collectively, tremendous progress has been made in the past decade in the understanding about the life cycle of AAV through the biophysical and structural studies. It is evident that the capsid structural dynamics plays central role throughout the viral infection and amplification process. Nonetheless, many key questions remain unaddressed, such as the exact mechanism triggering nucleus genome unloading and the high-resolution structure of the rep proteins in complex with the DNA and newly assembled capsid. The in-depth knowledge around the structural and functional properties of the capsid will aid the development of next generation of AAV vectors for gene therapy.

## Data Availability

Not applicable.

## References

[1] Samulski RJ, Muzyczka N (2014). AAV-mediated gene therapy for research and therapeutic purposes. Annual Rev Virol.

[2] Duan D (2018). Systemic AAV micro-dystrophin gene therapy for Duchenne muscular dystrophy. Mol Ther.

[3] Mijanović O, Branković A, Borovjagin AV, Butnaru DV, Bezrukov EA, Sukhanov RB, Ulasov I (2020). Battling neurodegenerative diseases with adeno-associated virus-based approaches. Viruses.

[4] Rodrigues GA, Shalaev E, Karami TK, Cunningham J, Slater NK, Rivers HM (2019). Pharmaceutical development of AAV-based gene therapy products for the eye. Pharm Res.

[5] Hasbrouck NC, High KA (2008). AAV-mediated gene transfer for the treatment of hemophilia B: problems and prospects. Gene Ther.

[6] Salabarria SM, Nair J, Clement N, Smith BK, Raben N, Fuller DD, Corti M (2020). Advancements in AAV-mediated gene therapy for Pompe disease. J Neuromuscul Dis.

[7] Colella P, Ronzitti G, Mingozzi F (2018). Emerging issues in AAV-mediated in vivo gene therapy. Mol Ther Methods Clin Dev.

[8] Kuzmin DA, Shutova MV, Johnston NR, Smith OP, Fedorin VV, Kukushkin YS, van der Loo JC (2021). The clinical landscape for AAV gene therapies. Nat Rev Drug Discov.

[9] Korneyenkov MA, Zamyatnin AA (2021). Next step in gene delivery: modern approaches and further perspectives of AAV tropism modification. Pharmaceutics.

[10] Asokan A, Schaffer DV, Samulski RJ (2012). The AAV vector toolkit: poised at the clinical crossroads. Mol Ther.

[11] Agbandje-McKenna M, Kleinschmidt J (2011) AAV capsid structure and cell interactions. Adeno-associated virus: methods and protocols. 47–92 10.1007/978-1-61779-370-7_3 22034026

[12] Naumer M, Sonntag F, Schmidt K, Nieto K, Panke C, Davey NE, Kleinschmidt JA (2012). Properties of the adeno-associated virus assembly-activating protein. J Virol.

[13] Weitzman MD, Kyöstiö SR, Kotin RM, Owens RA (1994). Adeno-associated virus (AAV) Rep proteins mediate complex formation between AAV DNA and its integration site in human DNA. Proc Natl Acad Sci.

[14] Berns KI (2020). The unusual properties of the AAV inverted terminal repeat. Hum Gene Ther.

[15] Srivastava A, Mallela KM, Deorkar N, Brophy G (2021). Manufacturing challenges and rational formulation development for AAV viral vectors. J Pharm Sci.

[16] Dhungel BP, Bailey CG, Rasko JE (2021). Journey to the center of the cell: tracing the path of AAV transduction. Trends Mol Med.

[17] Balakrishnan B, Jayandharan R (2014). G. Basic biology of adeno-associated virus (AAV) vectors used in gene therapy. Current Gene Ther..

[18] Xie Q, Bu W, Bhatia S, Hare J, Somasundaram T, Azzi A, Chapman MS (2002). The atomic structure of adeno-associated virus (AAV-2), a vector for human gene therapy. Proc Natl Acad Sci.

[19] Xie Q, Yoshioka CK, Chapman MS (2020). Adeno-associated virus (AAV-DJ)—cryo-EM structure at 1.56 Å resolution. Viruses.

[20] Lerch TF, Xie Q, Chapman MS (2010). The structure of adeno-associated virus serotype 3B (AAV-3B): insights into receptor binding and immune evasion. Virology.

[21] Govindasamy L, DiMattia MA, Gurda BL, Halder S, McKenna R, Chiorini JA, Agbandje-McKenna M (2013). Structural insights into adeno-associated virus serotype 5. J Virol.

[22] Ng R, Govindasamy L, Gurda BL, McKenna R, Kozyreva OG, Samulski RJ, Agbandje-McKenna M (2010). Structural characterization of the dual glycan binding adeno-associated virus serotype 6. J Virol.

[23] Nam HJ, Lane MD, Padron E, Gurda B, McKenna R, Kohlbrenner E, Agbandje-McKenna M (2007). Structure of adeno-associated virus serotype 8, a gene therapy vector. J Virol.

[24] DiMattia MA, Nam HJ, Van Vliet K, Mitchell M, Bennett A, Gurda BL, Agbandje-McKenna M (2012). Structural insight into the unique properties of adeno-associated virus serotype 9. J Virol.

[25] Kronenberg S, Kleinschmidt JA, Böttcher B (2001). Electron cryo-microscopy and image reconstruction of adeno-associated virus type 2 empty capsids. EMBO Rep.

[26] Kronenberg S, Böttcher B, von der Lieth CW, Bleker S, Kleinschmidt JA (2005). A conformational change in the adeno-associated virus type 2 capsid leads to the exposure of hidden VP1 N termini. J Virol.

[27] Mietzsch M, Jose A, Chipman P, Bhattacharya N, Daneshparvar N, McKenna R, Agbandje-McKenna M (2021). Completion of the AAV structural atlas: serotype capsid structures reveal clade-specific features. Viruses.

[28] Mietzsch M, Broecker F, Reinhardt A, Seeberger PH, Heilbronn R (2014). Differential adeno-associated virus serotype-specific interaction patterns with synthetic heparins and other glycans. J Virol.

[29] Xie Q, Spear JM, Noble AJ, Sousa DR, Meyer NL, Davulcu O, Zhang F, Linhardt RJ, Stagg SM, Chapman MS (2017). The 2.8 Å electron microscopy structure of adeno-associated virus-DJ bound by a heparinoid pentasaccharide. Mol Ther Methods Clin Dev.

[30] Huang LY, Patel A, Ng R, Miller EB, Halder S, McKenna R, Asokan A, Agbandje-McKenna M (2016). Characterization of the adeno-associated virus 1 and 6 sialic acid binding site. J Virol.

[31] Afione S, DiMattia MA, Halder S, Di Pasquale G, Agbandje-McKenna M, Chiorini JA (2015). Identification and mutagenesis of the adeno-associated virus 5 sialic acid binding region. J Virol.

[32] Zhang R, Cao L, Cui M, Sun Z, Hu M, Zhang R, Rao Z (2019). Adeno-associated virus 2 bound to its cellular receptor AAVR. Nat Microbiol.

[33] Zhang R, Xu G, Cao L, Sun Z, He Y, Cui M, Lou Z (2019). Divergent engagements between adeno-associated viruses with their cellular receptor AAVR. Nat Commun.

[34] Meyer NL, Hu G, Davulcu O, Xie Q, Noble AJ, Yoshioka C, Chapman MS (2019). Structure of the gene therapy vector, adeno-associated virus with its cell receptor. AAVR eLife.

[35] Xu G, Zhang R, Li H, Yin K, Ma X, Lou Z (2022). Structural basis for the neurotropic AAV9 and the engineered AAVPHP. eB recognition with cellular receptors. Mol Ther Methods Clin Dev..

[36] Hu G, Silveria MA, Zane GM, Chapman MS, Stagg SM (2022) Adeno-Associated Virus Receptor-Binding: Flexible Domains and Alternative Conformations through Cryo-Electron Tomography of Adeno-Associated Virus 2 (AAV2) and AAV5 Complexes. J Virol 96(13):00106–22 10.1128/jvi.00106-22 PMC9278096 35674430

[37] Padron E, Bowman V, Kaludov N, Govindasamy L, Levy H, Nick P, Agbandje-McKenna M (2005). Structure of adeno-associated virus type 4. J Virol.

[38] Hamilton BA, Wright JF (2021) Challenges posed by immune responses to AAV vectors: addressing root causes. Front Immunol 12:675897 10.3389/fimmu.2021.675897 PMC8168460 34084173

[39] Gurda BL, DiMattia MA, Miller EB, Bennett A, McKenna R, Weichert WS, Nelson CD, Chen WJ, Muzyczka N, Olson NH, Sinkovits RS (2013). Capsid antibodies to different adeno-associated virus serotypes bind common regions. J Virol.

[40] McCraw DM, O’Donnell JK, Taylor KA, Stagg SM, Chapman MS (2012). Structure of adeno-associated virus-2 in complex with neutralizing monoclonal antibody A20. Virology.

[41] Tseng YS, Gurda BL, Chipman P, McKenna R, Afione S, Chiorini JA, Agbandje-McKenna M (2015). Adeno-associated virus serotype 1 (AAV1)- and AAV5-antibody complex structures reveal evolutionary commonalities in parvovirus antigenic reactivity. J Virol.

[42] Jose A, Mietzsch M, Smith JK, Kurian J, Chipman P, McKenna R, Agbandje-McKenna M (2019). High-resolution structural characterization of a new adeno-associated virus serotype 5 antibody epitope toward engineering antibody-resistant recombinant gene delivery vectors. J Virol.

[43] Stahnke S, Lux K, Uhrig S, Kreppel F, Hösel M, Coutelle O, Ogris M, Hallek M, Büning H (2011). Intrinsic phospholipase A2 activity of adeno-associated virus is involved in endosomal escape of incoming particles. Virology.

[44] Girod A, Wobus CE, Zádori Z, Ried M, Leike K, Tijssen P, Kleinschmidt JA, Hallek M (2002). The VP1 capsid protein of adeno-associated virus type 2 is carrying a phospholipase A2 domain required for virus infectivity. J Gen Virol.

[45] Venkatakrishnan B (2012). Endosomal pH mediated structural transitions in adeno-associated viruses (Doctoral dissertation, University of Florida).

[46] Venkatakrishnan B, Yarbrough J, Domsic J, Bennett A, Bothner B, Kozyreva OG, Agbandje-McKenna M (2013). Structure and dynamics of adeno-associated virus serotype 1 VP1-unique N-terminal domain and its role in capsid trafficking. J Virol.

[47] Nam HJ, Gurda BL, McKenna R, Potter M, Byrne B, Salganik M, Muzyczka N, Agbandje-McKenna M (2011). Structural studies of adeno-associated virus serotype 8 capsid transitions associated with endosomal trafficking. J Virol.

[48] Penzes JJ, Chipman P, Bhattacharya N, Zeher A, Huang R, McKenna R, Agbandje-McKenna M (2021). Adeno-associated virus 9 structural rearrangements induced by endosomal trafficking pH and glycan attachment. J Virol.

[49] Yan Z, Zak R, Luxton GG, Ritchie TC, Bantel-Schaal U, Engelhardt JF (2002). Ubiquitination of both adeno-associated virus type 2 and 5 capsid proteins affects the transduction efficiency of recombinant vectors. J Virol.

[50] Mingozzi F, High KA (2011). Therapeutic in vivo gene transfer for genetic disease using AAV: progress and challenges. Nat Rev Genet.

[51] Gabriel N, Hareendran S, Sen D, Gadkari RA, Sudha G, Selot R, Hussain M, Dhaksnamoorthy R, Samuel R, Srinivasan N, Srivastava A (2013). Bioengineering of AAV2 capsid at specific serine, threonine, or lysine residues improves its transduction efficiency in vitro and in vivo. Hum Gene Ther Methods.

[52] Zhong L, Zhao W, Wu J, Li B, Zolotukhin S, Govindasamy L, Agbandje-McKenna M, Srivastava A (2007). A dual role of EGFR protein tyrosine kinase signaling in ubiquitination of AAV2 capsids and viral second-strand DNA synthesis. Mol Ther.

[53] Kelich JM, Ma J, Dong B, Wang Q, Chin M, Magura CM, Xiao W, Yang W (2015). Super-resolution imaging of nuclear import of adeno-associated virus in live cells. Mol Ther Methods Clin Dev.

[54] Rayaprolu V, Kruse S, Kant R, Venkatakrishnan B, Movahed N, Brooke D, Lins B, Bennett A, Potter T, McKenna R, Agbandje-McKenna M (2013). Comparative analysis of adeno-associated virus capsid stability and dynamics. J Virol.

[55] Horowitz ED, Rahman KS, Bower BD, Dismuke DJ, Falvo MR, Griffith JD, Harvey SC, Asokan A (2013). Biophysical and ultrastructural characterization of adeno-associated virus capsid uncoating and genome release. J Virol.

[56] Bernaud J, Rossi A, Fis A, Gardette L, Aillot L, Büning H, Castelnovo M, Salvetti A, Faivre-Moskalenko C (2018). Characterization of AAV vector particle stability at the single-capsid level. J Biol Phys.

[57] Sutter SO, Lkharrazi A, Schraner EM, Michaelsen K, Meier AF, Marx J, Vogt B, Büning H, Fraefel C (2022). Adeno-associated virus type 2 (AAV2) uncoating is a stepwise process and is linked to structural reorganization of the nucleolus. PLoS Pathog.

[58] Thomas CE, Storm TA, Huang Z, Kay MA (2004). Rapid uncoating of vector genomes is the key to efficient liver transduction with pseudotyped adeno-associated virus vectors. J Virol.

[59] Wistuba A, Weger S, Kern A, Kleinschmidt JA (1995). Intermediates of adeno-associated virus type 2 assembly: identification of soluble complexes containing Rep and Cap proteins. J Virol.

[60] Bennett A, Mietzsch M, Agbandje-McKenna M (2017). Understanding capsid assembly and genome packaging for adeno-associated viruses. Future Virol.

[61] Wörner TP, Bennett A, Habka S, Snijder J, Friese O, Powers T, Agbandje-McKenna M, Heck AJ (2021). Adeno-associated virus capsid assembly is divergent and stochastic. Nat Commun.

[62] Wu P, Xiao W, Conlon T, Hughes J, Agbandje-McKenna M, Ferkol T, Flotte T, Muzyczka N (2000). Mutational analysis of the adeno-associated virus type 2 (AAV2) capsid gene and construction of AAV2 vectors with altered tropism. J Virol.

[63] Sonntag F, Schmidt K, Kleinschmidt JA (2010). A viral assembly factor promotes AAV2 capsid formation in the nucleolus. Proc Natl Acad Sci.

[64] Maurer AC, Pacouret S, Diaz AKC, Blake J, Andres-Mateos E, Vandenberghe LH (2018). The assembly-activating protein promotes stability and interactions between AAV’s viral proteins to nucleate capsid assembly. Cell Reports.

[65] Prasad KMR, Zhou C, Trempe JP (1997). Characterization of the Rep78/adeno-associated virus complex. Virology.

[66] Bleker S, Pawlita M, Kleinschmidt JA (2006). Impact of capsid conformation and Rep-capsid interactions on adeno-associated virus type 2 genome packaging. J Virol.

[67] Gerlach B, Kleinschmidt JA, Böttcher B (2011). Conformational changes in adeno-associated virus type 1 induced by genome packaging. J Mol Biol.

